# Diet, Nutrition, and Oral Health: What Influences Mother’s Decisions on What to Feed Their Young Children?

**DOI:** 10.3390/ijerph18158159

**Published:** 2021-08-02

**Authors:** Amit Arora, Louise Chew, Kaye Kang, Lily Tang, Mohamed Estai, Jack Thepsourinthone, Navira Chandio, Jinal Parmar, Ashish M. Doyizode, Vipin Jain K., Sameer Bhole

**Affiliations:** 1 Campbelltown Campus, School of Health Sciences, Western Sydney University, Locked Bag 1797, Penrith, NSW 2751, Australia; 17538666@student.westernsydney.edu.au (J.T.); navira_c@yahoo.com (N.C.); jinalparmar3112@gmail.com (J.P.); dr.doyizode@gmail.com (A.M.D.); 2 Health Equity Laboratory, Campbelltown, NSW 2560, Australia; 3 Translational Health Research Institute, Western Sydney University, Locked Bag 1797, Penrith, NSW 2751, Australia; 4 Discipline of Child and Adolescent Health, Sydney Medical School, Faculty of Medicine and Health, The University of Sydney, Westmead, NSW 2145, Australia; 5 Oral Health Services, Sydney Dental Hospital, Sydney Local Health District, NSW Health, Surry Hills, NSW 2010, Australia; Sameer.Bhole@health.nsw.gov.au; 6 Sydney Dental School, Faculty of Medicine and Health, The University of Sydney, Surry Hills, NSW 2010, Australia; louise.ml.chew@gmail.com (L.C.); anymagous@yahoo.com (K.K.); lily.yihua.tang@gmail.com (L.T.); 7 Australian eHealth Research Centre, CSIRO, Floreat, WA 6014, Australia; Mohamed.Estai@csiro.au; 8 School of Human Sciences, University of Western Australia, Crawley, WA 6009, Australia; 9 Department of Public Health Dentistry, KLE’s Institute of Dental Sciences, Bangalore 560022, India; vipinjaink1@gmail.com

**Keywords:** preschool children, diet, food preferences, oral health, life course

## Abstract

The purpose of this study was to learn about mothers’ experiences with food choices for their pre-school children in underprivileged communities in Greater Western Sydney (GWS). A total of 20 mother-child dyads living in GWS were recruited to a qualitative study from an ongoing birth cohort study. Participants’ houses were visited for semi-structured interviews, which were recorded, transcribed verbatim, and analysed thematically. The interviews yielded five main themes: (i) food choices, nutrition, and health; (ii) accessibility and availability of foods (iii) buying time for parents; (iv) child’s age and their preference on food choices; (v) conditioning certain behaviours by family and cultural factors. Nutrition literacy, child’s preferences, unhealthy food intake by family members, child’s demand, advertising and availability of harmful foods, and time constraints were all mentioned as hurdles to mothers making appropriate meal choices for their children. However, some identified facilitators were promoting parents’ knowledge, increasing access to health educational materials, upskilling mothers to providing healthier alternatives, regulating the marketing of unhealth foods. Although, the present study identified critical factors that influence mothers’ food choices for their young children, making healthy food choices is a complex practice as it is shaped by individual, social and environmental influences.

## 1. Introduction

Early childhood caries (ECC) is a severe and progressive form of tooth decay that affects the primary teeth of children under the age of 6 years [1]. Despite the significant progress in child’s oral health, ECC remains the most prevalent chronic disease in young children [2]. The 2016 Global Burden of Disease Study reported that oral diseases globally affect at least 3.58 billion people, with 486 million children experience dental caries in their primary teeth worldwide [2]. The rates of ECC are the highest among the socially disadvantaged groups, such as those from a lower socio-economic status (SES), refugees, and ethnic minorities [3,4]. If left untreated, ECC may results in severe pain, abscesses, and may adversely impact children’s general health, growth, and quality of life [5,6]. Furthermore, due to the age of the child and severity of the disease, hospitalisation may be required to enable invasive dental treatment under general anaesthesia, which can be expensive [7]. This poses an additional financial burden on the healthcare system and causes significant distress to both families and young children.

Likewise, obesity attributed to 8.4% of the total disease burden among Australian population in 2015 [8]. According to 2017 Australian Institute of Health and Welfare report, overweight and/or obesity were prevalent among 20% of Australian children of aged 2–4 years [9]. Alarmingly, the burden of overweight and obesity is shared disproportionately: prevalence in lowest SES quintile children is higher than in highest SES quintile children [10]. Children with overweight/obesity are at higher risk of chronic diseases and overweight/obesity in adulthood [11], and recent evidence highlights that being overweight in early childhood leads to significantly higher costs [12]. Childhood overweight and obesity increases the risk of type 2 diabetes, cardiovascular disease and depression [13].

Recent research highlights that early childhood dietary behaviours play a significant role in dental caries and obesity development [14,15]. Fisher-Owens et al. proposed a multilevel conceptual model of children’s oral health, and suggested that ECC results from the interaction between individual, family, and community-level factors [16]. There is wealth of evidence that environmental, cultural, and socioeconomic determinants impact ECC and obesity development [14,17,18]. In particular, SES, parental practices, and home environment are essential family-level predictors of hygiene and diet-related conditions such as ECC and childhood obesity [14,19].

Parents, particularly mothers, make choices about health-related practice and offer an environment that supports the development of health-related behaviours. Furthermore, mother’s health behaviours are influenced by her knowledge, literacy, beliefs, culture and educational background [18]. Whilst children tend to adopt the behaviour of their parents [20], a mother’s feeding practices play a fundamental role in shaping the child’s dietary preferences and can influence her child’s risk of ECC and childhood obesity [14,21]. 

A wide range of psychosocial and behavioural factors affecting children’s diet preference are well-recognised [19,22]. Several factors linked to parental attitudes, practices, and SES are well-known to impact the dietary practices of young children [23,24,25,26,27,28,29]. However, in-depth qualitative evidence regarding the influence of broader social and family interactions, and environmental attributes on mothers’ food choices during early childhood are more limited [30]. 

There is a need to gain a holistic understanding of different influences on food choices made for young children, especially those living in disadvantaged areas, in order for primary preventive strategies are to be successful. Although high sugar intake and parental feeding practices are important risk factors, in-depth qualitative evidence underpinning unhealthy dietary behaviours early in the life course need to be better understood. A deeper understanding of influences on food choices may be derived from a qualitative research, particularly for socially deprived communities as they are under-represented in oral health research and share the highest oral disease burden. The present study aimed to explore facilitators and barriers that socially disadvantaged mothers experienced when establishing healthy dietary patterns for young children early in the life course and identifying their implications on a child’s oral health. 

## 2. Materials and Methods

### 2.1. Background

This qualitative study was nested within the Healthy Smiles Healthy Kids (HSHK) study (n = 1035) [31], a population-based cohort study that recruited mothers with newborn children living in Greater Western Sydney (GWS). The overall purpose of the HSHK study was to assess the relationship between early childhood feeding practices, dental health, and obesity in preschool children. 

### 2.2. Ethical Considerations

Ethics approval was obtained from the Human Research Ethics Committees of the former Sydney South West Area Health Service—RPAH Zone (ID number X08-0115), Western Sydney University, and the University of Sydney. This research has been conducted in full accordance with the World Medical Association Declaration of Helsinki. Written consent was obtained from all study participants.

### 2.3. Research Design

A qualitative approach was adopted using semi-structured interviews for data collection. This method was chosen mainly for two reasons. First, it has been shown to be useful in gaining an understanding of the complex social phenomena [32] and providing detailed information, particularly in disadvantaged communities [33]. Second, the study design is flexible and allows further investigation, information gathering, and analysis of new or emerging data/themes [34]. Furthermore, the health belief model was used in the study [35], which allows exploring the health-related behaviours.

The method and reporting of results were according to the Consolidated Criteria for Reporting Qualitative Research (COREQ) [36], the commonly used checklist in qualitative studies (Table S1).

### 2.4. Sampling

A purposive sampling technique was used as it enriches the quality of data collected via the selection of cases strategically and purposefully [37]. In this study, the investigators used the maximum variation sampling strategy to select a range of cases to allow variations on the areas of interest [38,39]. The investigators continued recruitment of participants until data saturation was achieved for the study; that is, all dimensions of interest were explored, and there was no new information required from more participants [40]. 

From the HSHK study, 20 mother-child dyads living in GWS were selected for a home interview from postcodes considered as disadvantaged according to the Australian 2006 Socio-Economic Index for Areas (SEIFA) rankings [41]. Mothers were invited via phone calls to participate and received an information pack containing participant information sheet, consent form, and a letter in the post informing them that they would be contacted and interviewed (in-depth) at their home regarding their child’s diet. 

To ensure that the participants represent a sample from a broad perspective, mothers for this nested study were:

(i) Either primiparous or multiparous;
(ii) From a range of education levels;
(iii) Either employed (skilled/unskilled) or unemployed and/or pensioners;
(iv) From a diverse ethnic background but able to speak in English.

### 2.5. Semi-Structured Interviews

Four researchers (A.A., L.C., K.K., L.T.) who had extensive experience in public health and in qualitative research conducted the interviews. The face-to-face interviews with mothers were conducted at their homes for approximately one hour. Discussion topics for semi-structured interviews were developed from our preliminary investigations and other studies [31,42]. Table 1 lists the interview guide that was used for discussion. The interviewers simultaneously made observations and recorded field notes of each interview.

All interviews were digitally recorded, immediately debriefed, and transcribed verbatim later. Interview debriefing served to evaluate data collection, summarise the main findings, and helped to prepare for subsequent interviews.

### 2.6. Data Analysis

Thematic analysis, a method for identifying, analysing, and reporting patterns within data [43] were used to analyse the data. Data were analysed using an iterative process involving reading and re-reading transcripts to reveal areas of interest or themes. In this study, analysis was undertaken in several phases.

The initial coding was developed by the lead investigator after reviewing all the transcripts using NVivo 9 (QSR International, Cambridge, MA, USA) qualitative data management and analysis software. Developing codes and categories is a common approach in qualitative data analysis [44] and informs the researcher about the data before making analytic interpretations. In the next phase, three researchers independently reviewed the data for manual coding and analysis. The researchers worked through each transcript systematically and identified concepts offered by the participants. This phase, not only confirmed the Phase 1 results but also gave credibility to the study findings [45]. Finally, all researchers then reviewed and compared the results of the NVivo coding and the independent manual coding analysis. All researchers reached a consensus on any discrepant categorization through discussion. Similar patterns of quotes were identified and grouped under the same theme [43]. Furthermore, the interaction between themes and subthemes were presented as a thematic map (Figure S1).

### 2.7. Rigour

In this study, several methodological strategies were adopted to ensure rigour. The in-depth interviews were conducted by four researchers having extensive experience in population oral health and qualitative interviewing. To evaluate data collection, summarisation of the main findings, and for the preparation of subsequent interviews, the interviews debriefing was consistently undertaken between the researchers until data saturation was reached. The audio-recorded interview’s verbatim transcriptions were carried out by professional transcription services to ensure accuracy. To enhance the credibility of the study findings, the transcriptions of the interviews were shared with the participants to seek clarification on the interpretation. For accuracy, the data coding was performed by four researchers independently; hence, to achieve team consensus. Moreover, to enhance rigour negative case analysis was undertaken. In the results section, along with comprehensive information on the study participants, and collected data, the participant’s direct quotes have been presented. Hence, the rigour criteria (i.e., dependability, credibility, confirmability, and transferability) to ensure trustworthiness in qualitative research have been addressed, by the above strategies [46,47]. 

### 2.8. Researcher Positionality

The acknowledgment of the researcher’s self-reflection along with subjective viewpoints makes the intellectual and personal biases explicit; hence, enhances the credibility of qualitative research findings [48]. In this study, the researcher’s ontological position was defined as relativist which precluded the researcher’s influence on study participants. For example, the participants were recruited by independent persons. The researcher’s philosophical position allows the prevention of the researcher’s ethnocentrism throughout the study period from interview conduction to data analysis. Furthermore, data analysis was conducted by following iteration steps (see above).

## 3. Results

A study participant response rate was 100%, as all the invited mothers (n = 20) consented to participate and completed the interviews. The mean age of the mothers was 31.3 ± 6.9 years, and half the participants’ (50%) were born in Australia. Only half the participants (50%) held university qualifications, and 60% were highly socio-economically disadvantaged. More than half the mothers (55%) were housewives, or unskilled labourers or in clerical roles. The mean age of the child was 30.1 ± 5.8 months and all of them were living with their parents with 30% having ECC. The demographic characteristics of the study’s participants are presented in Table 2.

Regarding meal choices, mothers reported that it varied from sandwiches to rice, pasta, meat, and vegetables. While reporting about drink choices, they mentioned that water was the main beverage in young children followed by milk and juices. Additionally, mothers reported that a variety of food items were consumed as snacks. The most consumed items were snacks including fresh fruit, yoghurt, and biscuits. Other snacks included cheese sticks, crackers, chips, nuts, dried fruit, and vegetable sticks. 

On examining the key influences in their food and drink choices, five themes emerged from the interviews: (i) Food choices, nutrition, and health; (ii) Accessibility and availability of foods (iii) Buying time for parents; (iv) Child’s age and their preference on food choices; (v) Conditioning certain behaviours by family and cultural factors. The themes and sub-themes are presented in Table 3.

### 3.1. Food Choices, Nutrition, and Health 

Health concerns and self-perceived nutritional values were the most important identified factors behind the choice of meals mothers made. Some mothers had knowledge of processed and unprocessed foods. When enquired about this knowledge, the most frequently indicated sources were family, friends, the internet, pamphlets, books, and health care professionals. Mothers also reported that they were concerned that poor dietary habits would not only affect their dental health, but also, increase the risk of obesity, diabetes, high cholesterol, and lack of concentration.

However, nutrition literacy was identified as the common barrier to healthy dietary choices. Mothers often reported having a general or low level of nutrition literacy. For instance, one mother explained that:

*“Anything that is not processed is a good start. Things that are not too high in sugar, not too high in fat, whole grains we can. Ahmmm…, and a variety of food… And um, I don’t buy things which are, which I know are really high in fats, or really have sugar.”*

(31-year-old mother from China)

Additionally, some mothers reported reading food labels but, generally, stated that they did not understand the nutrition labels and, hence, did not bother reading them when purchasing items at the supermarket. Often, they reported that when they purchased snacking products, the decision was based on the taste of the product rather than the nutritional value. 

Several mothers stated that they actively make informed choices while selecting their meals, drinks, and snacks, based on nutritional values. For instance, most of them believed that water was the best drink for their children as it was perceived as a source of hydration, while others reported specifically choosing tap water because of the fluoride.

*“The best drink is water. Everyone knows that it’s best for hydrating and it has fluoride to protect their teeth.”*

(31-year-old mother from South Africa)

Furthermore, mothers often reported giving their children sugar-sweetened beverages (predominantly fruit juices) as it was perceived to have health benefits such as vitamins. Some mothers preferred to dilute the fruit juice to lower the sugar content or used “a straw” to reduce the damage to their child’s teeth. Mothers also felt that milk drinks provide some health benefits such as calcium.

*“She has milk (in the mornings). Sometimes she will have the chocolate milk. I guess it is better than drinking coke… At least, she is getting some calcium.”*

(18-year-old mother from Australia)

### 3.2. Availability and Accessibility of Foods

Mothers also reported that the ease of access and availability of foods and drinks influenced their meal choices. This included the availability of food and drinks within the home, at the stores, and elsewhere. For instance, some mothers reported their preference to buy meals at stores, while others explained that they would bring food and drinks (e.g., sandwiches, juices) with them when going out. Some mothers reported that if children were at the shopping centre, they would want to eat cake or ice cream as snacks. However, if they were at home, the choice would depend on whatever is available around the household at the time. Other influences on food and drink choices included when they were away from home, such as in childcare, when dining out, or at social events such as birthdays. One mother commented on the availability of drinking water:

*“You can always get a cup of water from somewhere.”*

(28-year-old mother from Australia)

Interestingly, some mothers preferred to buy items that were on weekly specials at the supermarkets and decide the child’s diet based on what was currently on special. They reported that the weekly savings were important as they helped them to save money for the family. For instance, one mother stated:

*“Yeah, of course… we can’t afford to buy healthy food… I have a big family so weekly savings are important to us.”*

(49-year-old mother from Iraq)

Similarly, another mother explained individual financial situations to be a barrier to accessing a variety of healthy food and drink options:

*“Like the people who earn more money they have a variety. They wouldn’t be really budgeting for the meal. Like the people who are less fortunate. They budget so they wouldn’t really have a big range of what to choose from.”*

(28-year-old mother from India)

As such, mothers often described choosing cheaper food and drink options for their children. This included home brand products, fatty and/or low nutrition items, and fast food. For instance:

*“The cost of fast food and easy food fills up the tummy and is not expensive.”*

(31-year-old mother from India)

The location of stores also influenced food and drink choices. Some mothers reported that they spent a few hours going to their preferred shops to buy food and drink items.

### 3.3. Buying Time for Parents 

Barriers towards healthy eating included the time constraints in food preparation, abundant availability of unhealthy foods, food packaging, and advertising aimed directly at children. 

Time constraints in food preparation were also discussed as an influence on the meal, drink, and snack choices. For instance:

*“I work 3 days, my husband works full day, full week. And so, on any workday, coming home and being in a bit of a hurry to prepare, perhaps we don’t make smart food choices.”*

(31-year-old China)

Additionally, some mothers reported giving snacks to their children to engage whilst a meal was being prepared.

Mothers reported different patterns of snacking in their children. In some households, meals, drinks, and snacks were at set times—most commonly for breakfast, morning tea, lunch, afternoon tea, and dinner. In other households, children would eat and drink whenever they felt hungry or thirsty or would even snack throughout the day as a habit. One of them explains the spontaneity in their child’s eating/drinking habits:

*“She eats snacks in the afternoon, she gets more hungry then. In the morning, sometimes she won’t have a snack, sometimes she does. It’s basically when she asks for food.”*

(30-year-old mother from Australia)

The time of day was also reported to be an indicator of the types of food and drinks their child consumes. It was often reported that access to drinks such as milk was often at set times of the day, such as breakfast. Similarly, access to fruit juice was most common with meals or with morning tea. Other drinks frequently consumed by children during this time included flavoured milk and fruit smoothies.

### 3.4. Child’s Age and Their Preference on Food Choices

Mothers often indicated that age was an influencing factor on the children’s food and drinks choices. One who had older children felt that it was acceptable for an older child to drink sugar-sweetened beverages while expressing that younger child should predominantly be drinking water or milk. They also stated that, as children grow older, it was more acceptable to give them other drink choices.

*“I guess when she goes to school, she will drink soft drinks like her brother. But she is too young to have all that sugar from the drinks.”*

(29-year-old mother from Australia)

Conversely, some mothers reported less concern for their younger children as they believed that health concerns were of significance at an older age. One mother commented:

*“I will buy chips and biscuits weekly… He likes the taste. He is still too young to worry about any health problems, so I don’t worry about it much”*

(38-year-old mother from Australia)

Some mothers expressed that sugar and fat content were not significant when the child was young and physically active.

*“I do look at the fat content but that’s more for us (parents), I don’t really look at the fat content for her.”*

(29-year-old mother from India)

Children were often reported to have autonomy in their meal, drink, and snack options and that their taste preferences often varied with age. It, however, was commonly reported that their child preferred sweeter foods. Most mothers reported that their children preferred drinks that tasted sweet. Certain textures of food items were also influential in their child’s consumption of food. For instance:

*“I wouldn’t say he is a fussy eater … he wouldn’t really eat mushy type textures. It would make him gag—avocado, mashed potato, banana. It was the texture, not the flavour at all.”*

(28-year-old mother from Australia)

A few of them in this study reported that they rarely prepared snacks for children. Often, children themselves decided their food choices or were influenced by those around them or the food consumed by other household members.

*“She opens the fridge and gets it… she comes, and she wants me to wash it for her. She knows when she’s hungry and she wants it.”*

(26-year-old mother from Lebanon)

Mothers reported that the children knew about the snacking products through media advertisements, friends, or at supermarkets. They also reported that if they refused to purchase the snacking items that their child demanded, it was common for them to feel embarrassed if the child started showing tantrums at the supermarkets.

*“She sees things at the shops with bright colours and cartoons and she gets attracted to it. If I refuse to purchase, she starts having a tantrum at the supermarket, so I am forced to buy sometimes as I feel embarrassed…”*

(18-year-old mother from Australia)

Some mothers recommended that manufacturers should not make certain foods attractive as it encouraged children to eat unhealthy foods.

*“Yesterday we went shopping together and she wanted to buy some of those xx things, and I said no… She doesn’t know what flavoured yoghurt is, she’s never tried it. It’s colourful. It’s got toys or Dora on it. She sees it and she want it.”*

(30-year-old mother from Australia)

### 3.5. Conditioning Certain Behaviours by Family and Cultural Factors

Mothers reported family and cultural background to influence their child’s meal, drink, and snack consumption. Grandparents, aunts, uncles, and older siblings were indicated to be major influences. Grandparents, in particular, were stated to “spoil” their child with unhealthy food and drink options. Furthermore, the cultural background and culinary skills of the mother were reported to predominantly influence the preparation style of the child’s meals. For instance:

*“We’re Lebanese so we use a lot of wheat in our cooking, and chicken, just like everybody else, but the way how we do it it’s different so that’s how I cook at home.”*

(26-year-old mother from Lebanon)

Additionally, some mothers reported that their child’s dietary choices were based on their previous experiences with the older children.

*“When my (older) son who was young, he used to always drink from a bottle. I’d reckon that’s probably why he got a lot tooth decays. So, with my (younger) son, usually I prefer him to make him drink from a straw because I’ve heard that’s better. Passes your teeth, so I’d choose that way.”*

(28-year-old mother from Australia)

Mothers often reported employing various strategies to condition particular behaviours and dietary habits within their child(ren). To encourage healthy eating, mothers reported employing certain strategies such as hiding healthy food items amongst ones that the child likes, offering rewards, verbal positive reinforcement (e.g., “yum”), distracting the child, trickery, playing games, and encouraging the child to eat healthy foods. Treats were commonly given as rewards for good behaviour, or achievements. These treats were usually sweet in nature, including lollies (candies) and chocolate. Other rewards included giving additional television or leisure time if the child was obedient (e.g., ate their vegetables). Mothers also stated that children should be encouraged to eat and drink healthy in childhood as they would adopt similar habits later in life.

*“As a treat, it’s nice but I am not going to get him into the habit. I think if he learns that it’s a treat, then it’s fine.”*

(29-year-old mother from Australia)

Moreover, in order to reduce the consumption of fatty, low nutrition, and/or sugar-sweetened food and drinks, mothers reported that they would lie to the child that the food or drink was a medicine, distasteful, or that they would keep these foods and drinks away from the sight of children. A few also reported not buying them in the first instance.

*“I just avoid buying them (sugar-sweetened beverages) … If I have to buy when our friends are visiting, I will hide them in the pantry and serve in special glasses so they (children) can’t see what is in the glass.”*

(28-year-old mother from India)

However, some mothers find it difficult to say no when their child asks for a particular food item or drink. Others, on the other hand, do not have a problem as they feel it is their duty to reduce their child’s consumption of unhealthy foods.

*“I think that it’s your responsibility as a parent to kind of give your kids the best start you can… If I’m tired, my resistance is lower. So, you know, after a working day coming home … I’m more likely to give in to what they’re asking for. Whereas in the morning or on a non-workday, I’m very confident to just to what I’ve said I know is the right choice.”*

(31-year-old mother from South Africa)

As a rule, mothers thought that role modelling behaviour was really important in influencing their child’s meal, drink, and snack choices. Mothers reported that young children are influenced by siblings and parents and that they used role modelling to promote healthy food and drink consumption. For instance:

*“The kids always want to stealS my drink so I’d rather drink water.”*

(38-year-old mother from Australia)

## 4. Discussion

Since feeding habits are established in early childhood, the failure to implement healthy dietary practices in children may contribute to a higher rate of ECC. The present study identified key content themes that provide essential insights into what needs to be addressed to establish healthy feeding practices during early childhood. Findings demonstrated that nutrition literacy, knowledge, beliefs, child’s preference, and accessibility and affordability of healthy foods were perceived as major influences on food choices. In addition, the child’s autonomy in making food choices, difficulty in withstanding the child’s temperament, advertising aimed at children, time constraints in preparing healthy meals, and abundant availability of unhealthy foods were also key influences. 

To our knowledge, the current study is the first research that explored socially disadvantaged mothers’ experience of making healthy food choices for preschool children in Australia. Boak et al. examined the experiences of families from regional and rural Australia on making food choices for their infants [30]. Their study revealed that beliefs, knowledge, infants’ own preferences, parent’s capacity, and cost and availabilities of various foods were key influences on food choices for infants [30]. Since individuals and their environments are connected, understanding the influence of individual factors encompassed by the social and environmental circumstances can explain how food choices are made for children. 

Health and nutrition literacy was often regarded as a major barrier to make healthy food choices. Although most mothers claimed confidence regarding their perceptions of healthy foods, they seemed to be unaware of the diet-related health risks associated with high sugar intake. For instance, mothers commonly offered diluted fruit juices to their children, which were often diluted with water, and sweet treats were used as rewards for shaping children’s behaviour. Similar findings were reported in previous studies [49,50]. The World Health Organization (WHO) guidelines strongly recommend children and adults to reduce their daily intake of free sugars to <5% of their total intake [51]. In addition, mothers reported having low nutrition literacy, as they had difficulty in understanding nutritional information on labels of snaking products. Failure to check the sugar contents in food labels has been observed in other studies where most mothers were unaware of the sugar content in most food items [52,53]. 

A mother’s selection of foods is critical to children’s long-term dietary and feeding behaviours, and this can be influenced by parents’ attitudes, culture, and beliefs. Parents’ beliefs can affect children’s oral health in other ways; children of parents with a poor attitude to oral hygiene or healthy food had higher rates of dental caries [54]. For example, some mothers felt that providing sweets between meals acted as a source of energy, while others believed it was acceptable for older children to drink sweetened beverages. Although not all cultural practices have negative impacts, it is important for the health teams to identify and educate mothers in a culturally appropriate manner to avoid adverse practices that could potentially affect their child’s oral health [55]. 

Children’s autonomy on their food and their preferences may influence the food that their parents provide. Mothers would often base their children’s meals on food costs, the product’s taste, or child preference, consistent with findings from other studies [49,50,56]. Children often reported having autonomy in their drinks and meals, which may explain the difficulty in denying the child’s demands. Furthermore, parental feeding practices were found to be associated with a child’s temperament, with difficult children more likely to experience higher rates of dental caries than easy children [57]. A few mothers also reported that their food choices were often compromised by the influence of other family members, which was reported in other studies [52,58]. These parental factors must be considered while developing interventions to promote a child’s oral health.

Lack of knowledge about healthy foods may not be the only significant barrier to healthy food choices among low SES mothers. Accessibility and affordability of healthy foods may affect a mother’s choices more than knowledge. Petrunoff et al. demonstrated more similarities than differences between low SES and high SES mothers concerning knowledge of healthy food [49]. Lower SES communities with limited access to quality fresh food at affordable prices relied on consuming energy-dense, nutrient-poor take-away foods [59]. It is well-recognised that there is a higher density of delivery food stores and lower availability of fresh products in lower SES regions. The increased supply of healthy foods and health messages to increase the consumption of healthy foods could be a solution to increase the choice of healthier options for disadvantaged families [60].

Time constraints and work pressure were other barriers perceived in making healthy food choices for children. Previous studies in the USA, Canada, and Australia indicated that mother’s lifestyles, particularly a lack of time and conflicting family priorities, often made it challenging to ensure that their children had healthy diets [61,62,63]. Adopting durable health and feeding practices during early childhood should begin at home, as mothers play an important role in shaping the child’s health behaviours. Mothers are required to invest more time and be informed that their own feeding habits will significantly impact their children’s oral health and, consequently their quality of life. For instance, being a role model and emphasizing healthy food messages in cooking, eating together, and having patience while introducing new foods have been effective strategies [64]. 

Food advertising aimed directly at children was indicated as a barrier to healthy food choices for children. Persuasive marketing techniques, such as promotional and licensed characters, influence children’s food preferences and snack selection [65,66,67]. An Australian study found that food and/or drink advertisements were aired more frequently during children’s television viewing hours compared to other viewing hours [68]. Direct marketing and strategic EDNP food placement have also raised concerns before [49]. There should be more restrictions on displaying unhealthy foods in retailers and regulating advertisements that are aimed directly at children. 

### 4.1. Strengths and Limitations

This study has number of identifiable strengths. The use of a qualitative approach helps to explore an in-depth understand of facilitators and barriers mothers were experiencing while establishing a healthy dietary pattern for young children. Furthermore, the study design flexibility allowed to collect and analyse study data simultaneously; hence provided opportunities to gathered additional information [69]. This study, as a part of HSHK birth cohort study, and all the invited mothers consented for participation; allowed to achieve a 100% participants response rate. The study sample size (20 mothers) was adequate to achieve data saturation, which means all dimensions of interest were explored, and there was no new information required from more participants [69]. 

The present study has some limitations. Despite adequate sample size, the data collected from the participants were representative of their influence on the child’s diet, leaving little scope for adequate representation of other attributes (e.g., father, childcare, or other family members). There is also a possibility that perception and social desirability may introduce bias. The data on mothers’ health behaviours, for example, oral hygiene behaviours and dietary practices were not collected as part of this study. 

### 4.2. Implications and Recommendations

Several barriers and facilitators based on the experiences of the socially disadvantaged mothers while establishing healthy dietary patterns for young children were identified. Most frequently reported facilitators included mothers’ knowledge of healthy foods and nutrition values, increase access to oral health educational materials, rewards for good behaviour, or achievements, mothers’ healthy decisions to obscuring unhealthy foods from sights of the children, and the role modelling strategies, whereas common barriers included inadequate health and nutrition literacy of mothers, availability, costs, and affordability of unhealthy foods, time constraints, family members influence, advertisement of unhealthy foods, and child autonomy (Table 3). 

These study findings suggest certain recommendations for policy makers and health professionals. While designing ECC preventive programs, consideration should be given to the social characteristics of the family, diet and feeding practices, and how the socio-behavioural risk factors of ECC may be modified. Furthermore, the preventive strategies targeting mothers or caregivers should consider increasing access to health educational leaflets, nutrition literacy campaigns, and empowering and upskilling mothers to withstand children’s demands. The health policies promoting the intake of healthier foods and reducing the high sugar intake should be promoted; these may include taxation and limiting the advertising of unhealthy foods and drinks.

## 5. Conclusions

The present study reported a wide range of key factors that provide essential and pragmatic additional insights into what needs to be addressed to ensure healthy dietary practices in early childhood. Making healthy food choices appears to be a complex practice as it is shaped by the individual, social, community, and environmental influences experienced by mothers and families. 

## Figures and Tables

**Table 1:** Semi-structured interview guide.

What do you think is a healthy and unhealthy diet? Why? How important is it that your child eats and drinks healthy? Why? What are the sources of information you rely on for information on healthy eating? How do you decide on the food choices in your family? How do you decide the drinks your child drinks? What do you think about fruit juices? How do you decide the snack your children have? What makes it easier for you to feed your child the right kinds of food? What makes it difficult for you to feed your child the right kinds of food?

**Table 2:** Characteristics of the study participants.

	n (%)	M	SD
Mother’s age (years)		31.27	6.92
Child’s age (months)		30.11	5.82
Child’s sex			
Male	10 (50%)		
Female	10 (50%)		
Number of Children			
One	11 (55%)		
Two or more	09 (45%)		
Early Childhood Caries			
Yes	06 (30%)		
No	14 (70%)		
Mother’s Country of Birth			
Australia	10 (50%)		
China	03 (15%)		
India	02 (10%)		
Middle East	03 (15%)		
Africa	02 (10%)		
IRSAD			
Least Disadvantaged	01 (5%)		
Low Disadvantaged	07 (35%)		
Highly Disadvantaged	12 (60%)		
Mother’s education			
<Year 12	07 (35%)		
College	03 (15%)		
University	10 (50%)		
Employment			
Home Duties	04 (20%)		
Manager and Professional	09 (45%)		
Clerical	05 (25%)		
Unskilled Labour	02 (10%)		

M—Mean, SD—Standard Deviation, IRSAD—Index of Relative Socio-Economic Disadvantage.

**Table 3:** Summary of themes with facilitators and barriers segregated subthemes.

Themes	Subthemes
**Theme 1. Food choices, nutrition and health**	**Facilitators** Self-perceived nutrition value of foods Mother’s health literacy including knowledge of healthy foods Multiple sources of nutritional information **Barriers** Inadequate health and nutrition literacy of mothers.
**Theme 2. Availability and accessibility of foods**	**Facilitators** Easily available heathy food items, while dinning out or other places of outing. **Barriers** Availability, costs, and affordability of unhealthy foods.
**Theme 3. Buying time for parents**	**Facilitators** Empowering and upskilling mothers to withstand children demands. **Barriers** Time constraints in the preparation of healthy meals. Abundant availability of unhealthy foods and advertising aimed directly at young children.
**Theme 4. Child’s age and their preference on food choices**	**Facilitators** Put restrictions on advertising unhealthy foods on TV and social media. Restrict the display of unhealthy products in retailers. **Barriers** Children autonomy in making choices for their meals and drinks. Mothers’ assumption of an association between choice of food and children age Inability to withstand the child’s demand and temperament. Role of family members, friends, and media to influence children feeding choice
**Theme 5. Conditioning certain behaviours by family and cultural factors**	**Facilitators** Previous experience with older children influences mother’s dietary choices for younger child. Encourage rewards for good behaviour, or achievements. Mothers’ healthy decisions to obscure unhealthy foods from sights of the children Role modelling behaviour influences child’s meal, drink, and snack choices. **Barriers** Culture, belief, and misconception about healthy foods.

## Data Availability

The data presented in this study are not publicly available due to privacy.

## Supplementary Materials

The following are available online at https://www.mdpi.com/article/10.3390/ijerph18158159/s1, Table S1: COREQ (COnsolidated criteria for REporting Qualitative research) Checklist, Figure S1: Thematic map showing the interaction between themes and subthemes.
