# Supplementary material for: Diet, Nutrition, and Oral Health: What Influences Mother’s Decisions on What to Feed Their Young Children?
Source: Int J Environ Res Public Health. 2021 Aug 2;18(15):8159. doi: 10.3390/ijerph18158159 (PMC8345989; doi:10.3390/ijerph18158159)
Supplement: Supplementary file 1 [file ijerph-18-08159-s001.zip › ijerph-1298408 supplementary.pdf]

Supplementary Material

Table S1. COREQ (COnsolidated criteria for REporting Qualitative research) Checklist.

Item No. and Topic	Guide Questions/Description	Response and Reported on Page No.
**Domain 1: Research team and reflexivity**		
Personal characteristics		
1. Interviewer/facilitator	Which author/s conducted the interview or focus group?	A.A., L.C., K.K., and L.T (Page 3)
2. Credentials	What were the researcher's credentials? E.g. PhD, MD	A.A.: MPH, PhD, Senior Lecturer<br>L.C. : BDent<br>K.K.: BDent<br>L.T.: BDent
3. Occupation	What was their occupation at the time of the study?	Research students and researchers
4. Gender	Was the researcher male or female?	One male and 3 females
5. Experience and training	What experience or training did the researcher have?	Interviewers had prior experience in qualitative interviewing and population oral health (Page 3). Other researchers are involved in other research projects.
Relationship with participants		
6. Relationship established	Was a relationship established prior to study commencement?	A.A. had recruited the participants for birth cohort sudy on child oral health. Other researchers had no relationships with the participants before the study.
7. Participant knowledge of the interviewer	What did the participants know about the researcher? e.g. personal goals, reasons for doing the research	Participants knew about the reasons for doing research as indicated in the participant information sheets (Page 3).
8. Interviewer characteristics	What characteristics were reported about the inter viewer/facilitator? e.g. Bias, assumptions, reasons and interests in the research topic	Apart from their names and affiliation, participants did not know about interviewers' characteristics.
**Domain 2: Study design**		
Theoretical framework		
9. Methodological orientation and Theory	What methodological orientation was stated to underpin the study? e.g. grounded theory, discourse analysis, ethnography, phenomenology, content analysis	Qualitative Research Design and Health Belif Model (Page 3)
Participant selection		
10. Sampling	How were participants selected? e.g. purposive, convenience, consecutive, snowball	Purposive sampling technique (Page 3)
11. Method of approach	How were participants approached? e.g. face-to-face, telephone, mail, email	Telephone (Page 3)
12. Sample size	How many participants were in the study?	20 mothers (Page 5)

13. Non-participation	How many people refused to participate or dropped out? Reasons?	None refused to participate or dropped out
	Setting	
14. Setting of data collection	Where was the data collected? e.g. home, clinic, workplace	Home interviews (Page 3)
15. Presence of non-participants	Was anyone else present besides the participants and researchers?	Only the participants and interviewers were present (Page 3)
16. Description of sample	What are the important characteristics of the sample? e.g. demographic data, date	The demographic characteristics of the study participants are outlined in Table 2 (Page 5)
	Data collection	
17. Interview guide	Were questions, prompts, guides provided by the authors? Was it pilot tested?	Semi-structured interview guide is presented in Table 1. It was also pilot tested (Page 4).
18. Repeat interviews	Were repeat interviews carried out? If yes, how many?	Repeat interviews were not carried out
19. Audio/visual recording	Did the research use audio or visual recording to collect the data?	Interviews were audio-recorded (Page 3)
20. Field notes	Were field notes made during and/or after the interview or focus group?	Field notes were made immediately after each interview and they helped in data analysis.
21. Duration	What was the duration of the interviews or focus group?	One hour (Page 3)
22. Data saturation	Was data saturation discussed?	Yes (Page 3)
23. Transcripts returned	Were transcripts returned to participants for comment and/or correction?	Yes (Page 4)
	**Domain 3: Analysis and findings**	
	Data analysis	
24. Number of data coders	How many data coders coded the data?	4 researchers- A.A., L.C., K.K., and L.T. (Page 4)
25. Description of the coding tree	Did authors provide a description of the coding tree?	Yes (Figure S1)
26. Derivation of themes	Were themes identified in advance or derived from the data?	Themes were derived from the data (Page 4)
27. Software	What software, if applicable, was used to manage the data?	NVivo 9 software (Page 4)
28. Participant checking	Did participants provide feedback on the findings?	Yes
	Reporting	
29. Quotations presented	Were participant quotations presented to illustrate the themes/findings? Was each quotation identified? e.g. participant number	Yes, participant quotations were presented (Page 7-10). Quotations were de-identified for confidentiality reasons.

30. Data and findings consistent	Was there consistency between the data presented and the findings?	Yes, as indicated in Rigor (Page 4)
31. Clarity of major themes	Were major themes clearly presented in the findings?	Yes, presented in Results (Page 5-10)
32. Clarity of minor themes	Is there a description of diverse cases or discussion of minor themes?	Yes, presented in Discussion (Page 10-11)

Developed from: Tong A, Sainsbury P, Craig J. Consolidated criteria for reporting qualitative research (COREQ): a 32-item checklist for interviews and focus groups. *International Journal for Quality in Health Care*. 2007. Volume 19, Number 6: pp. 349 – 357.

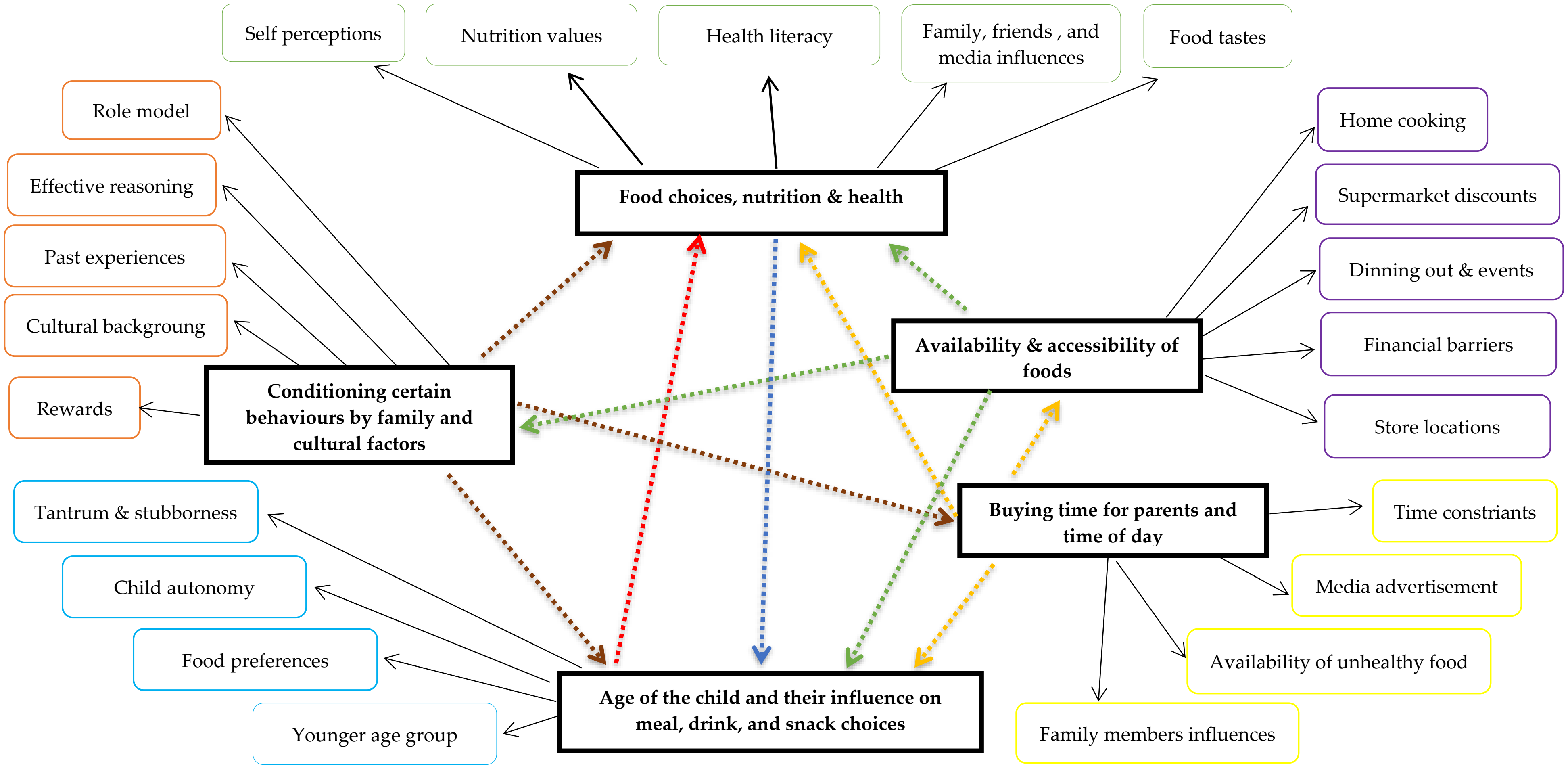

Figure S1. Thematic map showing the interaction between themes and subthemes.
